# Nab-paclitaxel-based compared to docetaxel-based induction chemotherapy regimens for locally advanced squamous cell carcinoma of the head and neck

**DOI:** 10.1002/cam4.382

**Published:** 2015-01-26

**Authors:** Amy Schell, Jessica Ley, Ningying Wu, Kathryn Trinkaus, Tanya Marya Wildes, Loren Michel, Wade Thorstad, Hiram Gay, James Lewis, Jason Rich, Jason Diaz, Randal C Paniello, Brian Nussenbaum, Douglas R Adkins

**Affiliations:** 1 Division of Oncology, Department of Internal Medicine, Washington University School of MedicineSt. Louis, Missouri; 2 Division of Biostatistics, Washington University School of MedicineSt. Louis, Missouri; 3 Alvin J Siteman Cancer Center, Washington University School of MedicineSt. Louis, Missouri; 4 Department of Radiation Oncology, Washington University School of MedicineSt. Louis, Missouri; 5 Division of Surgical Pathology, Department of Pathology and Immunology, Washington University School of MedicineSt. Louis, Missouri; 6 Department of Otolaryngology-Head & Neck Surgery, Washington University School of MedicineSt. Louis, Missouri

**Keywords:** Docetaxel, head and neck cancer, induction chemotherapy, *nab*-paclitaxel, p16

## Abstract

We previously reported that *nab*-paclitaxel-based induction chemotherapy (IC) and concurrent chemoradiotherapy resulted in low relapse rates (13%) and excellent survival in head and neck squamous cell carcinoma (HNSCC). We compare the disease-specific survival (DSS) and overall survival (OS) between patients given *nab*-paclitaxel, cisplatin, and fluorouracil with cetuximab (APF-C) and historical controls given docetaxel, cisplatin, and fluorouracil with cetuximab (TPF-C). Patients with locally advanced HNSCC were treated with APF-C (*n *=* *30) or TPF-C (*n *=* *38). After 3 cycles of IC, patients were scheduled to receive cisplatin concurrent with definitive radiotherapy. T and N classification and smoking history were similar between the two groups and within p16-positive and p16-negative subsets. The median duration of follow-up for living patients in the APF-C group was 43.5 (range: 30–58) months versus 52 (range: 13–84) months for TPF-C. The 2-year DSS for patients treated with APF-C was 96.7% [95% Confidence Interval (CI): 85.2%, 99.8%] and with TPF-C was 77.6% (CI: 62.6%, 89.7%) (*P *=* *0.0004). Disease progression that resulted in death was more frequent in the TPF-C group (39%) compared with the APF-C group (3%) when adjusted for competing risks of death from other causes (Gray's test, *P *=* *0.0004). In p16 positive OPSCC, the 2-year DSS for APF-C was 100% and for TPF-C was 74.6% (CI: 47.4%, 94.6%) (*P *=* *0.0019) and the 2-year OS for APF-C was 94.1% (CI: 65.0%, 99.2%) and for TPF-C was 74.6% (CI: 39.8%, 91.1%) (*P *=* *0.013). In p16 negative HNSCC, the 2-year DSS for APF-C was 91.7% (CI: 67.6%, 99.6%) and for TPF-C was 82.6% (CI: 64.4%, 94.8%) (*P *=* *0.092). A 2-year DSS and OS were significantly better with a *nab*-paclitaxel-based IC regimen (APF-C) compared to a docetaxel-based IC regimen (TPF-C) in p16-positive OPSCC.

## Introduction

Most patients with squamous cell carcinoma of the head and neck (HNSCC) present with locally advanced disease (stage III–IV), and 5-year overall survival (OS) rates with multimodality treatment are 40–60% 1. Induction chemotherapy (IC) may be employed as a treatment strategy for locally advanced HNSCC, as it has the advantages of potential organ preservation, early identification of patients likely to benefit from chemoradiotherapy (CRT), and decreased incidence of distant metastases. The most widely used IC regimen is docetaxel, cisplatin, and fluorouracil (TPF), based on two randomized trials that showed a survival benefit with docetaxel added to cisplatin and fluorouracil (PF) 2,3.

*Nab*-paclitaxel is a microtubule inhibitor formulated as a colloidal suspension of paclitaxel and human serum albumin. Preclinical studies suggest that albumin binding to cell surface receptors can facilitate transport of *nab*-paclitaxel into tumor cells 4. We recently published results of a prospective phase 2 trial investigating a novel induction regimen of *nab*-paclitaxel, cisplatin, and fluorouracil with cetuximab (APF-C), followed by CRT 5. Survival outcomes in patients treated with APF-C followed by CRT were very favorable, with a relapse rate of only 13%.

We hypothesized that a *nab*-paclitaxel-based compared to a docetaxel-based IC regimen would be better in terms of survival endpoints. We compared survival outcomes of patients treated with APF-C to institutional historical controls who received TPF plus cetuximab (TPF-C). Both groups were subsequently scheduled to receive CRT with cisplatin.

## Materials and Methods

### Patient population

All patient data were collected through an institutional review board (IRB)-approved retrospective analysis. Patients treated with APF-C (*n *=* *30) were enrolled onto an IRB-approved prospective phase 2 trial at our institution between 2009 and 2010 (clinicaltrials.gov NCT00736944). The results of this trial were published, but were updated for this analysis 5. The historical comparison group consisted of patients who received IC with TPF-C (*n *=* *38) and were enrolled onto an IRB-approved head and neck registry between 2006 and 2010. Eligibility criteria for both IC groups included untreated Stage III and IVa/b HNSCC, Eastern Cooperative Oncology Group (ECOG) performance status of 0–1, T2–4 classification, and plan to receive three cycles of IC followed by cisplatin concurrent with definitive RT.

### Treatment regimens

#### Induction chemotherapy

The APF-C group was treated with every 3-week cycles of IV *nab*-paclitaxel 100 mg/m^2^ on days 1, 8, and 15, cisplatin 75 mg/m^2^ on day 1, 5-FU 750 mg/m^2^ continuous IV infusion (CIVI) daily on days 1–3, and cetuximab 400 mg/m^2^ day 1 and 250 mg/m^2^ weekly subsequently 5. The TPF-C group was treated with every 3-week cycles of docetaxel 75 mg/m^2^ and cisplatin 75 mg/m^2^ on day 1, 5-FU 750 mg/m^2^ CIVI daily on days 1–3, and cetuximab 400 mg/m^2^ day 1 and 250 mg/m^2^ weekly subsequently. Three cycles of IC were planned for both groups.

#### Definition of primary tumor site response criteria

Response at the primary tumor site was determined by visual analysis by the otolaryngologist after 2 cycles of IC. Response was categorized as complete response (CR: complete resolution of the primary tumor), partial response (PR: greater than 50% decrease but less than CR), stable disease (SD: 0–49% decrease) or progressive disease (PD: any increase), as previously described 5. Patients with favorable response (CR/PR) at the primary site proceeded to CRT after cycle 3 of IC. Surgical intervention was considered in patients with an unfavorable (SD/PD) tumor response at the primary site.

#### Chemoradiotherapy

CRT was started after the final cycle of IC. Intensity-modulated radiation therapy (IMRT) was administered once daily, 5 days weekly, as previously described 5. The APF-C and TPF-C groups received cisplatin 100 mg/m^2^ on day 1, 22, and 43 if creatinine <2.0 (if not, cetuximab was administered, as previously described) 5.

### Standard assessments

Baseline assessments included history and physical examination, laryngoscopy, computed tomography (CT) of the neck, and F-18 fluorodeoxyglucose positron emission tomography/CT (FDG-PET/CT). Assessments of adverse events (AEs) were performed using National Cancer Institute–Common Toxicity Criteria (NCI-CTC) version 3.0. After two cycles of IC, patients underwent assessment of tumor response by clinical examination and CT neck 5. Subsequent follow-up was performed as previously reported 5. Comorbidities were quantified using the Adult Comorbidity Evaluation (ACE)-27 index 6.

#### p16

Immunohistochemistry for p16, a surrogate for human papillomavirus (HPV), was performed on oropharyngeal (OP) tumors, as previously described 7 and was scored by the pathologist (JSL) as positive when ≥50% of tumor cells showed nuclear and cytoplasmic staining.

### Statistical methods

Demographic and disease characteristics of the patients were summarized using descriptive statistics. Treatment differences regarding response rates at the primary tumor site were examined using Cochran–Mantel–Haenszel, and Fisher's exact test for unordered or fewer than 3 categories and Jonckheere tests for ordinal trend for 3 or more categories. Disease-specific survival (DSS) was estimated by the cumulative incidence method to account for competing risks of death due to treatment-related or other causes. Cox proportional hazards models were used to estimate cause-specific hazards.

OS was defined as time from diagnosis to death or to last follow-up alive. Progression-free survival (PFS) was defined as time from diagnosis to death due to disease progression, to disease progression, or to last follow-up alive. DSS was defined as time from diagnosis to death from disease or to last follow-up alive.

## Results

### Patient and tumor characteristics

A number 68 patients were included in this analysis: 30 in the APF-C group and 38 in the TPF-C group. Baseline characteristics including T&N classification, smoking history, and primary tumor site were similar between the two groups (Table1). A larger proportion of patients in the APF-C group (57%) had p16-positive OPSCC in comparison to the TPF-C group (34.1%) (*P *=* *0.068). Therefore, survival outcome analyses were stratified for this important prognostic variable. Importantly, in either the p16-positive or p16-negative subsets, there were no significant differences in the proportions of patients who smoked, who had T2, T3, or T4 classification, or who had N0/1, N2 or N3 classification in the APF-C compared to the TPF-C groups (data not shown).

**Table 1:** Patient and tumor characteristics

	APF-C (*n *=* *30)		TPF-C (*n *=* *38)		*P* value
	No.	%	No.	%	
Characteristic					
Age (years)					
Median	57		55		0.95^2^
Range	38–71		38–72		
Sex					
Male	28	93	34	90	0.69^3^
Female	2	7	4	10	
Smoking history					
Yes	27	90	35	92	0.99^3^
No	3	10	3	8	
ACE comorbidity index					
0 (none)	9	30	9	24	0.12^4^
1 (mild)	2	7	17	45	
2 (moderate)	13	43	9	24	
3 (severe)	6	20	3	8	
Primary site					
Oropharynx	22	73	25	66	0.57^3^
Larynx	7	23	12	32	
Hypopharynx	0	0	1	3	
Oral cavity	1	3	0	0	
T classification					
T2	8	27	5	13	0.27^4^
T3	11	37	16	42	
T4	11	37	17	45	
N classification					
N0 and N1	6	20	10	26	0.83^4^
N2a/b	6	20	9	24	
N2c	14	47	10	26	
N3	4	13	9	24	
p16 positive					
Oropharynx^1^	17	57	13	34	0.068^3^

1 Two patients with oropharynx HNSCC did not have p16 staining performed and are not included in these data. Overall, there were 47 patients with oropharynx tumors, 30 of whom were p16 positive and were included here.

2 *P*-value from a *t*-test.

3 Fisher's exact test.

4 Jonckheere–Terpstra test (for trend over an ordinal variable).

### Treatment delivery

Treatment delivery details are described in Table2. Here 100% of the APF-C and the TPF-C groups completed 2 or more IC cycles, 97% of APF-C, and 84% of TPF-C patients received 3 IC cycles.

**Table 2:** Treatment delivery

Treatment	APF-C (*n *=* *30)		TPF-C (*n *=* *38)	
	% Total dose (median, range)	No. (%) receiving <100% of total dose	% Total dose (median, range)	No. (%) receiving <100% of total dose
Induction chemotherapy				
*nab*-Paclitaxel	100 (67–100)	14 (47)	–	–
Docetaxel	–	–	100 (67–100)	11 (29)
Cetuximab	100 (27–100)	14 (47)	89 (0–100)	21 (57)
Cisplatin	100 (67–100)	2 (7)	100 (67–100)	8 (21)
Fluorouracil	100 (80–100)	2 (7)	100 (58–100)	14 (37)
Chemoradiotherapy		1		2
Radiation therapy	*n *=* *29		*n *=* *34	
Median dose Gy (range)	70 (14–72)	3 (10)	70 (70––72)	4 (11)
Median elapsed days (range)	50 (8–69)		50 (37–64)	
Concurrent chemotherapy	*n *=* *28		*n *=* *34	
Cisplatin (% patients)	27 (93)		29 (85)	
% Total dose (range)	76 (33–100)	17 (57)	71 (33–100)	27 (71)
Cetuximab (% patients)	1 (4)		5 (15)	

1 Denominator is 30 patients.

2 Denominator is 38 patients.

Twenty-nine of 30 (96.7%) patients given APF-C and 34 of 38 (89.5%) patients given TPF-C received IMRT. Reasons that patients did not receive IMRT included treatment-related mortality (TRM) during IC (*n *=* *2), metastatic disease progression occurring during comorbidity-related treatment delay (*n *=* *1), surgery following IC with no adjuvant therapy (*n *=* *1), and lost to follow-up (*n *=* *1). The IMRT delivery was similar across the two IC groups. The majority of patients in the APF-C (93%) and TPF-C (85%) groups received cisplatin concurrent with IMRT. Others received cetuximab concurrent with IMRT.

### Primary tumor site response after 2 cycles of IC

The overall response rate at the primary site after 2 cycles of IC was 100% for APF-C and 84% for TPF-C (*P *=* *0.031). Progressive or stable disease after 2 cycles of TPF-C was seen in 13% (*n *=* *5) and 3% (*n *=* *1) of patients, respectively. Patients treated with APF-C had a greater risk of overall response at the primary tumor site than those treated with TPF-C after adjusting for p16 status (relative risk = 1.17; 95% confidence interval [CI]: 1.02–1.34).

Overall, the CR rate at the primary site was 53.3% for APF-C compared to 34.2% for TPF-C (*P *=* *0.14). In p16-positive OPSCC, the CR rate at the primary tumor site was 64.7% for APF-C and 38.5% for TPF-C (*P *=* *0.27). In p16 negative HNSCC, the CR rate was 33.3% for APF-C and 33.3% for TPF-C (*P *=* *0.99).

### Neck nodal and overall tumor site response after 2 cycles of IC

The tumor response rates at neck nodal sites after 2 cycles of APF-C and TPF-C respectively, based on clinical examinations were 61% CR (11 patients) compared to 45% CR (13 patients) and 39% PR (7 patients) compared to 48% PR (14 patients). In the APF-C group, the neck nodes of all patients who were evaluated responded, but in the TPF-C group stable or progressive nodal disease was seen in 7% (2 patients). Twelve patients in the APF-C group and 9 patients in the TPF-C group were not evaluable for this endpoint because of initial absence of nodal disease on clinical examination.

The overall tumor response rates for APF-C were 43% CR, 57% PR, and 0% SD/PD, whereas the overall tumor response rates for TPF-C were 26% CR, 58% PR, and 16% SD/PD.

### Disease-specific survival

The median duration of follow-up for living patients in the APF-C group was 43.5 (range: 30–58) months versus 52 (range: 13–84) months for TPF-C. All but 4 of the living patients had a minimum of 2 years of follow-up.

**Figure 1:**
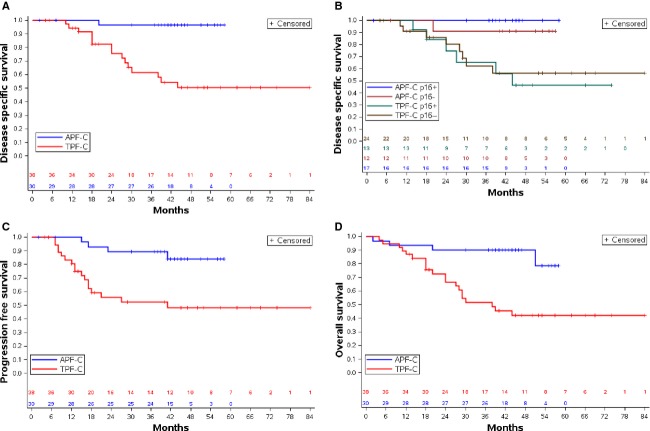
Survival outcomes in the APF-C and TPF-C groups. (A) DSS, (B) DSS by p16 status and treatment group, (C) PFS, (D) OS.

Two-year DSS was 96.7% (CI: 85.2%, 99.8%) for patients given APF-C compared to 77.6% (CI: 62.6%, 89.7%) for patients given TPF-C (*P *=* *0.0004) (Fig.1A). Table3 displays the univariate analysis of proportions of treatment failures due to disease for patient, tumor, and treatment characteristics. Given the prognostic significance of HPV in HNSCC 8, we stratified DSS for p16 status and treatment group. The 2-year DSS of patients with p16-positive OPSCC was 100% for APF-C (*n *=* *17) and 74.6% (CI: 47.4%, 94.6%) for TPF-C (*n *=* *13) (*P *=* *0.0019) (Fig.1B). Two-year DSS of patients with p16 negative HNSCC was 91.7% (CI: 67.6%, 99.6%) for APF-C (*n *=* *12) versus 82.6% (CI: 64.4%, 94.8%) for TPF-C (*n *=* *24) (*P *=* *0.092).

**Table 3:** Univariate analysis for DSS.

Variable	Failed/total no. patients	Log-rank *P* value
Patient characteristics		
Gender		
Male	16/62	0.99
Female	0/6	
Smoking		
Yes	15/62	0.59
No	1/6	
ACE comorbidity score		
None/mild	11/37	0.18
Moderate/severe	5/31	
Tumor characteristics		
Primary tumor site		
Oropharynx	11/47	0.93
Larynx/hypopharynx/oral cavity	5/21	
T classification		
T4	10/28	0.46 (T3 vs. T2) 0.095 (T4 vs. T2)
T3	5/27	
T2	1/13	
N classification		
N3	7/13	0.46 (N2 vs. N0/N1) 0.022 (N3 vs. N0/N1)
N2	7/39	
N0/1	2/16	
p16		
Negative	9/36	0.47
Positive	6/30	
Treatment characteristics		
CR at primary site		
No	12/39	0.24
Yes	4/29	
Treatment group		
TPF-C	15/38	0.0064
APF-C	1/30	

### Progression-free and overall survival

Overall, 2-year PFS was 89.3% for APF-C and 55.7% for TPF-C (*P *=* *0.0019) (Fig.1C). Two-year PFS for patients with p16-positive OPSCC was 93.8% for APF-C and 68.4% for TPF-C (*P *=* *0.034). The 2-year PFS for patients with p16 negative HNSCC was 81.8% for APF-C, and 51.2% with TPF-C (*P *=* *0.091).

Overall, 2-year OS was 90.0% for APF-C and 66.6% for TPF-C (*P *=* *0.0008) (Fig.1D). The 2-year OS for patients with p16-positive OPSCC was 94.1% for APF-C and 74.6% for TPF-C (*P *=* *0.013). The 2-year OS for patients with p16 negative HNSCC was 83.3% for APF-C and 65.4% for TPF-C (*P *=* *0.088).

### Causes of death

**Figure 2:**
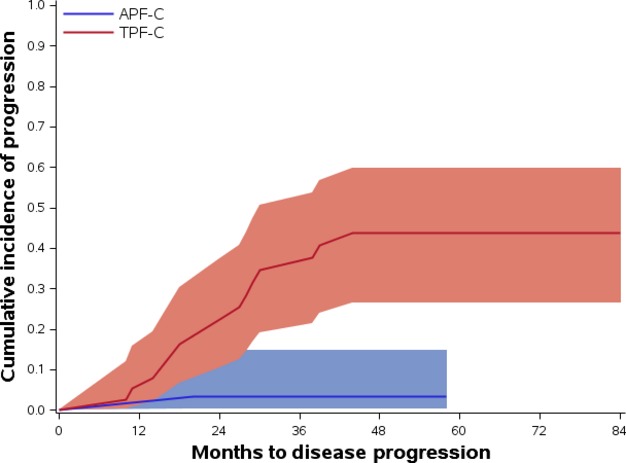
Cumulative incidence of death due to disease in the APF-C and TPF-C groups.

Causes of death are shown in Table4. Disease progression resulting in death was more common in the TPF-C group (39%) compared to the APF-C group (3%). A competing risks model (Gray's test) 9 was used to differentiate deaths due to disease progression from other causes of death without censoring deaths of other causes. This model showed that the incidence of death due to disease progression was greater in the TPF-C group compared to the APF-C group (*P *=* *0.0004). The hazard of death due to disease progression is 94% lower in the APF-C group than in the TPF-C group (HR = 0.060 [CI: 0.008, 0.45]). There was no difference in the hazard ratios of TRM or death of other causes (second malignancy and noncancer death) in the two treatment groups (TRM:HR = 0.632 [CI: 0.057, 6.97]); death of other causes [HR = 0.701 (CI: 0.116, 4.24)] (Fig.2).

**Table 4:** Cause of death and selected adverse events occurring with IC and CRT

	APF-C and CRT (*n *=* *30)	TPF-C and CRT (*n *=* *38)	*P* value^2^
	Number of patients (%)		
Cause of death			
Disease progression	1 (3)	15 (39)	–
Second malignancy	0 (0)	1 (3)	–
Treatment-related	1 (3)	2 (5)	–
Noncancer^1^	2 (6)	2 (5)	–
Total	4 (13)	20 (53)	–

Adverse event	Grade 3 or 4	All grades	Grade 3 or 4	All grades	
Neuropathy	1 (3)	17 (57)	1 (3)	4 (11)	0.00014
Hypersensitivity	1 (3)	4 (13)	2 (5)	11 (29)	0.15
Rash	6 (20)	28 (93)	5 (13)	29 (76)	0.096
Mucositis	9 (30)	25 (83)	13 (34)	26 (68)	0.26
Creatinine elevation	2 (7)	18 (60)	1 (3)	7 (18)	0.00085

1 Includes bowel obstruction (*n* = 1) and unknown (*n* = 2).

2 *P* value is for comparison of all grades of toxicities, *P*-values from Fisher's Exact test.

### Site of relapse and salvage surgery

Four patients (13%) in the APF-C group developed relapse (3 local/regional, 1 distant). In the TPF-C group, 17 patients (45%) developed relapse (8 local/regional, 5 distant, 4 both).

An unfavorable response to IC occurred in 6 patients (5 PD;1 SD); one of these patients underwent salvage surgery and 5 proceeded to CRT (patient/physician preference or not surgical candidate). Following CRT, 1 patient in the APF-C group underwent a neck dissection for a residual neck mass and the pathology was negative for malignancy. In the TPF-C group, 1 patient underwent post-treatment resection of residual malignancy.

### Adverse events

Table4 summarizes the AEs related to treatment during IC and CRT. Neuropathy and creatinine elevation were more frequent with APF-C, although most were grade 1–2.

## Discussion

This study is a historical comparison of survival outcomes of different taxane-based IC regimens in HNSCC. Patients treated with the *nab*-paclitaxel-based IC regimen (APF-C) followed by CRT had better 2-year DSS and OS and a lower hazard of death due to disease progression compared to the docetaxel-based regimen (TPF-C) followed by CRT. T and N classifications and smoking history were balanced between the two treatment groups and within p16-positive and p16-negative subsets. Differences in the proportion of patients with p16-positive tumors existed, favoring better outcomes in the APF-C group. However, it was within the p16-positive OPSCC subset that we observed significantly better 2-year DSS and OS with APF-C compared to TPF-C (DSS: 100% vs. 74.6%, *P *=* *0.0019 and OS: 94.1% vs. 74.6%, *P *=* *0.013, respectively).

Relapse of disease was more frequent in the TPF-C group compared to the APF-C group (Kaplan–Meier estimate 51.8% vs. 16%, respectively at 41 months). Given the mix of p16 negative HNSCC and p16-positive OPSCC patients, both of which were enriched for smoking history and bulky disease, the risk of relapse of disease was very low with APF-C. In the TAX 324 and Paradigm trials, relapse of disease occurred in 56% and 24% of patients given TPF followed by CRT, respectively 3,10,11. The higher risk of relapse of disease observed with TPF-C in comparison to TPF in the Paradigm trial may reflect the greater proportion of patients with T3/4 and N2c/3 classification in our study.

The 2-year PFS and OS were 89.3%, and 90%, respectively, for all patients treated with APF-C. Recognizing the potential pitfalls of cross-study comparisons, these findings compare very favorably to contemporary studies of TPF followed by CRT, with 2 or 3-year PFS rates of 54-67% and 2 or 3-year OS rates of 67–73% 10,11. Absolute PFS and OS with APF-C were better than with TPF in the TAX 324 trial in p16 negative HNSCC (2-year PFS 75% and 35%, respectively; 2-year OS 83.3% and 48%, respectively) and p16-positive OPSCC (2-year PFS 88.2% and 83%, respectively; 2-year OS 94% and 89%, respectively) 11. Cetuximab has been incorporated into docetaxel-containing IC, with 3-year PFS and OS of 70% and 74%, respectively 12.

Two questions arise about the relative contributions of components of APF-C. Is *nab*-paclitaxel better than cremophor-based paclitaxel in HNSCC and is there a role for cetuximab in IC? A comparison of cremophor-based paclitaxel and PF to PF showed no significant difference in 2-year OS (66.5% vs. 53.6%, respectively) 13. Although cross-study comparisons have limitations, the 2-year OS (90%) results with APF-C compare more favorably than these data. SPARC (secreted protein acidic and rich in cysteine) plays a role in albumin receptor-mediated endothelial transport 14. SPARC expression is common in tumor and stromal cells of HNSCC but not in adjacent normal oral mucosa 15, and correlated with tumor response to nab-paclitaxel in HNSCC in one study 4. Macropinocytosis, the process by which macromolecules like albumin are taken up into cells, is upregulated in the setting of activated RAS or PI3K pathways 16. RAS and/or components of the PI3K pathways are frequently activated in HNSCC 17–22, in particular p16-positive OPSCC, and could explain the high anti-tumor effect of *nab*-paclitaxel in HNSCC. Collectively, these data suggest but do not prove that *nab*-paclitaxel may be better than cremophor-based paclitaxel in HNSCC.

With respect to the second question, the EXTREME trial showed that the addition of cetuximab to chemotherapy improved tumor response rates and OS in patients with incurable HNSCC 23. Therefore, we anticipated an improvement in tumor response rates at the primary tumor site and 2-year survival outcomes with the addition of cetuximab to TPF and to APF. However, our trial was not designed to assess this hypothesis. We are prospectively evaluating nab-paclitaxel, cisplatin, and 5-fluorouracil (APF) without cetuximab as IC before CRT for HNSCC (NCT01566435) and plan to compare outcomes with the APF-C regimen.

IC is a controversial therapeutic strategy in HNSCC. While a few trials had shown an OS benefit with this approach 24,25, other trials have not confirmed this. The two largest trials evaluating the role of IC with CRT were the PARADIGM and DeCIDE trials 10,26. Both studies randomized patients to CRT alone or TPF followed by CRT. Neither trial reached its target accrual or showed an OS benefit with TPF. Both trials were limited by the fact that the preplanned 3-year OS was 50–55%; however, the actual OS was higher (73% and 75%), lowering the statistical power. The DeCIDE trial did show improvements in recurrence-free survival and distant failure rates with TPF.

Given these results, is there a justification for further investigation of IC in HNSCC? A rational reason to administer IC is to use the tumor response to IC as a method to stratify patients into higher and lower risk groups. CR at the primary tumor site to IC is an important predictor of long-term DFS 27. Those patients with a favorable response at the primary tumor site to IC could be candidates for deintensification of definitive therapy, and those with unfavorable response to IC may benefit from standard intensive therapy. This approach could substantially decrease the toxicity for lower risk patients. ECOG 1308 is a phase 2 trial investigating this novel reason to administer IC 28. In our study, the CR rate at the primary tumor site in p16-positive OPSCC was 64.7% with APF-C compared to 38.5% with TPF-C. Therefore, APF-C may be an excellent IC regimen to employ for risk stratification in p16-positive OPSCC.

Rates of AEs in our series were similar to that published for TPF, as well as IC regimens that incorporated cetuximab. In a large trial of TPF, the incidence of grade 3-4 events was 65% 2. IC regimens with cetuximab showed rates of mucositis (77%) and rash (45%) similar to APF-C 29. One TRM (3%) occurred in the APF-C group and 2 TRM (5%) in the TPF-C group. Peripheral neuropathy and creatinine elevation were more frequent in the APF-C group compared to the TPF-C group, although the majority of these AEs were grades 1–2.

Our study has limitations. This study was a retrospective analysis comparing outcomes in a prospective study with those of historical controls. The small sample size in each group limits the power of the statistical comparisons and the ability to control for heterogeneity in patient and tumor characteristics. We did not assess patient-reported quality of life in either treatment group. Also, cetuximab was included in both induction regimens; however, the role of cetuximab in this setting is unclear. This is a single institution experience that will require validation across institutions.

In conclusion, 2-year DSS and OS were significantly better with a *nab*-paclitaxel-based IC regimen (APF-C) compared to a docetaxel-based IC regimen (TPF-C) in p16-positive OPSCC. The outcomes support further research of *nab*-paclitaxel-based IC regimens in HNSCC.

## Conflict of Interest

None declared.
